# 

**Published:** 1834-10-01

**Authors:** 


					THE
MEDICAL
QUARTERLY REVIEW.
VOL. III.
LONDON:
J. SOUTER, 73, ST. PAUL'S CIIURCII-YARD;
SOLD ALSO IJV DlinOESS AND HILL; J. CHURCHILL; K. COX ; J. HIGHLEY J
IIEN81IAW ANT) UUSII, tVC.
W. JACKSON, NEW YOHK, AGENT 1011 THK UNITED STATES.
JT3 Qr1835-
w
1
lA
6
4
?
z
F
J. AND C. A D LARD, PRINTERS,
BARTHOLOMEW CLOSE.
NOTICES.
We have received " Observations on the Functional Affections of the Spinal
Cord," by Dr. and Mr. Griffin, and Dr. Hastings' " Account of the Natural
History of Worcestershire we shall review them, in our next.
tVc arc much obliged to Mr. Valentine for his suggestions, and shall take
them into consideration.
fVc regret that we cannot insert Dr. Balfour's Paper.
The Essay signed Ignis Fatuus is ingenious, but unsuited to a Medical
Journal: it shall be left for the author at our publisher's.

				

